# The inducible blockage of RNAi reveals a role for polyunsaturated fatty acids in the regulation of dsRNA-endocytic capacity in *Bactrocera dorsalis*

**DOI:** 10.1038/s41598-017-05971-0

**Published:** 2017-07-17

**Authors:** Xiaolong Dong, Xiaoxue Li, Qiujia Li, Hongmei Jia, Hongyu Zhang

**Affiliations:** 1 Key Laboratory of Horticultural Plant Biology (Ministry of Education), State Key Laboratory of Agricultural Microbiology, College of Plant Science and Technology, Huazhong Agricultural University, Wuhan, 430070 Hubei People’s Republic of China; 2 Institute of Medicinal Plant Development, Chinese Academy of Medical Sciences and Peking Union Medical College, No. 151 Malianwa North Road, Haidian District, Beijing 100193 People’s Republic of China

## Abstract

Exogenous double-stranded RNA (dsRNA) can trigger gene silencing through the RNA interference (RNAi) pathway. Our previous research established that *Bactrocera dorsalis* can block RNAi after an initial priming of exposure to dsRNA. However, the mechanism underlying this phenomenon is not yet fully understood. Here, we demonstrate that fatty acid biosynthesis and metabolism pathways play important roles in the blockage of RNAi induced by dsRNA priming. The ratio of linoleic acid (LA) to arachidonic acid (AA) was significantly increased in the hemolymph of *B. dorsalis* following dsRNA priming, and further, the endocytosis of dsRNA into the midgut cells of *B. dorsalis* was inhibited in these samples. The expression levels of most genes involved in the fatty acid biosynthesis and metabolism pathways were altered following priming with dsRNA. Furthermore, altering the composition of fatty acids via the injection of AA can facilitate the uptake of ingested dsRNA into the midgut cells of *Drosophila melanogaster* and successfully induce an RNAi effect, which cannot be achieved via feeding in fruit flies. Our results suggest that polyunsaturated fatty acids are involved in the regulation of the dsRNA-endocytic ability in *B. dorsalis*.

## Introduction

RNA interference (RNAi) is a conserved regulatory mechanism that is triggered by dsRNA; it functions in a remarkable variety of organisms^1^. As RNAi can be achieved easily in many eukaryote species, it has great potential in many areas of scientific research. RNAi is an efficient tool for fundamental research in molecular genetics. Genome-wide RNAi-based screens to identify genes involved in many biological processes have been performed^2^. Even though the clinical utility of RNAi has not yet been realized, ongoing RNAi-based preclinical and clinical trials still provide hope for success^3^. In agriculture, a number of studies have established that RNAi can be used as an environmentally-friendly pest management strategy. Two landmark studies demonstrate the feasibility of controlling pests by genetically modifying crops to express dsRNA^4, 5^.

Two mechanisms regulate the entery of dsRNA into cells. Studies in *Caenorhabditis elegans* reveals that *sid-1* gene encodes a transmembrane protein serving as a channel for import of dsRNA into cells and spreading of dsRNA throughout the animal^6^. The *sid-2* gene in *C. elegans* mediating the initial uptake of ingested dsRNA from the lumenal space via endocytosis^7^. However, in several insect genomes, including the best known model insect, *Drosophila. melanogaster*, no *sid* gene orthologs were found^8^. Studies using S2 cells have demonstrated that clathrin-mediated endocytosis is responsible for the uptake of dsRNA^9^. In *Bactrocera dorsalis*, entry of dsRNA into cells also depend on the clathrin-mediated endocytosis machinery^10^. After entering cells, dsRNA is processed into siRNAs of 21 to 25 bp long by the RNase III Dicer enzyme. These siRNAs are then loaded into the RNA-induced silencing complex and can recognize homologous mRNAs and trigger their degradation by RNase enzymes^11^. However, RNAi in insects appears varying results in different species; some insect species seem to be insenstive to dsRNA. For example, feeding dsRNAs to adult *D.melanogaster* failed to induce RNAi effect^12^. *B.dorsalis*, flies can block RNAi after an initial priming with dsRNA molecules that target endogenous genes^10^. So, it seems that there exist some mechanism regulating the endocytic ability of dsRNA to influence RNAi effect. However, this kind of mechanism remains unclear.

Membrane traffic, including endocytosis, requires two processes of membrane fusion, one of which is called fission^13^. Fission refers to the pinching-off of the vesicle from its donor membrane^14^. In clathrin-mediated endocytosis, the endocytic vesicles are encapsulated by a polygonal clathrin coat and formed with the aid of polymerization of the GTPase dynamin^15^. Membrane lipids are important to the clathrin-mediated endocytosis. For example, the heterotetrameric adaptor protein, AP2, which mediates clathrin assembly, binds to phosphoinositides in the membrane^16^. Membrane phosphoinositides, in particular, phosphatidylinositol-4,5-bisphosphate has also been implicated in vesicle-trafficking pathways via its impact on the cytoskeleton^17^.

In this study, we found that the dsRNA-induced loss of endocytic ability in *B.dorsalis* is mediated by polyunsaturated fatty acids (PUFAs). The expression of many genes involved in the fatty acid biosynthesis and metabolism pathways were altered after primary priming phase of feeding with dsRNA. Silencing of the key gene *fasn* could rescue endocytic ability of dsRNA in *B.dorsalis*. Furthermore, changing the composition of fatty acids by injecting arachidonic acid can successfully induce an RNAi effect in *D. melanogaster* by facilitating the uptake of ingested dsRNA molecules into midgut cells. Our results suggest that polyunsaturated fatty acids take part in the regulation of endocytosis of dsRNA in *B. dorsalis* and there may be an immune-like response in invertebrates that inhibits the entry of exogenous nucleic acids, and this response may result in abnormal gene expression in host cells.

## Results

### Hemolymph of ds-*rpl19* challenged flies can induce RNAi blocking

In our previous study we found that *B*. *dorsalis* can block RNAi effect after an initial priming with a dsRNA solution^10^. Insect hemolymph is not only considered as a depository of nutrients and energy, but also plays key roles in many physiological activites such as immune responses and substance transportation. Thus we hypothesized that there may exist some factors mediating the RNAi blocking in the hemolymph of *B*. *dorsalis*. To test our hypothesis, hemolymph transfer experiments were performed. Firstly, we induced the blockage to RNAi in *B.dorsalis*. Engineered bacteria was used to express dsRNA in the present study. After being extracted and purified, the dsRNA was tested by electrophoresis (Supplementary Figure S1A). There may exist a small amount of *E. coli* RNA in the dsRNA extracted from the bacteria. So the dsRNA concentrations obtained by NanoDrop measurements may be reflecting the total RNA concentrations in our research. However, the *E. coli* RNA mixed in desired dsRNA seemed to have no influence on the inducible RNAi blockage since ds-*egfp* and ds-*rpl19* were both prepared by bacteria and we also observed the same inducible RNAi blocking in *B.dorsalis* by using dsRNAs prepared by *in vitro* transcription (Supplementary Figure S1B). Flies in the challenged group (Ch) were fed with ds-*rpl19* as a primary exposure; in the naïve (Nv) group, ds-*egfp* was applied as a primary exposure. After secondary exposure to ds-*rpl19*, the Nv group showed efficient RNAi, and the mRNA accumulation of *rpl19* decreased by 59%. However, in the Ch group, after secondary exposure to ds-*rpl19*, depletion of *rpl19* could not be observed (Supplementary Figure S2A).

**Figure 1:**
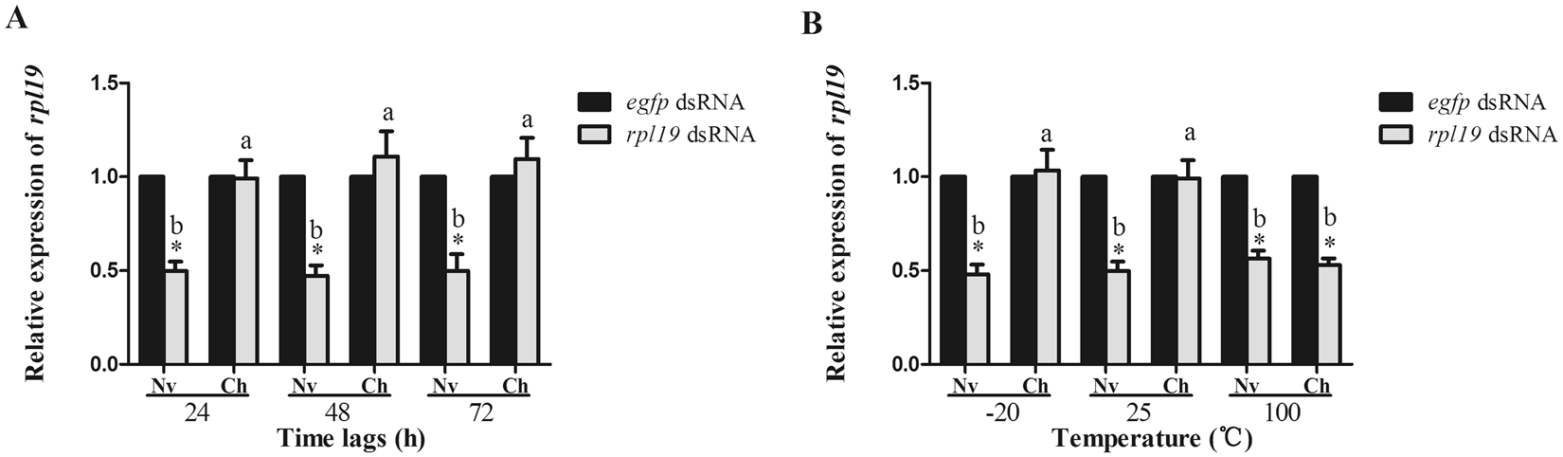
The blockage of RNAi in *B. dorsalis* can be induced by injecting hemolymph collected from dsRNA-challenged flies. **(A)** mRNA accumulation of the target gene after feeding ds*-rpl19* followed by injection of Ch hemolymph which has been incubated at 25 °C for 24 h, 48 h and 72 h. (**B**) mRNA accumulation of the target gene after feeding ds*-rpl19* followed by injecting hemolymph treated at different temperatures. Normalized target gene mRNA accumulation is reported relative to mRNA accumulation of the ds-*egfp* control, which was set to 1. All error bars represent the S.E. of the mean of three independent biological replicates. * indicates a statistically significant difference in *rpl19* mRNA accumulation between the feeding ds-*rpl19* group and the control ds-*egfp* group (P < 0.05, Student’s t-test). Different letters indicate a significant difference in *rpl19* mRNA accumulation among the feeding ds-*rpl19* treatments (P < 0.05, Duncan’s test).

Next, the hemolymph of Nv and Ch flies was collected and injected into untreated flies. After injecting hemolymph that had been incubated at 25 °C for 24 h, 48 h, and 72 h, flies were fed with ds-*rpl19*. As expected, the mRNA level of *rpl19* in flies injected with hemolymph from the Nv group had decreased of 51%, 53%, and 50% after feeding, respectively. However, depletion of *rpl19* was not observed in flies injected with hemolymph from the Ch group (Fig. 1A). Low temperature treatment (−20 °C for 24 h) had no effect on the ability of the hemolymph to block RNAi. Injecting the hemolymph, which had been treated at 100 °C for 10 min, could not influence the RNAi effect (Fig. 1B).

### Metabolomic analysis of hemolymph from Ch flies

Comparative metabolomics analysis using ultra performance liquid chromatography mass spectrometry (UPLC/MS) was performed to explore the possible small molecules that may be related to the observed induction of RNAi blocking in the hemolymph (Supplementary Figure S2B). We identified 37 metabolites displaying at different levels between the two sample groups (Supplementary Table S1). A model obtained in an orthogonal projection to latent structures–discriminant analysis (OPLS-DA) showed clear differences between the metabolomes of the Nv and Ch hemolymph samples. The data points were clustered into two distinct groups in the plot map, indicating obvious separation between the Nv and Ch groups (Supplementary Figure S2C–S2D).

Overall, several kinds of metabolites displayed considerable differences between Ch and Nv hemolymph, including multiple lipids, carbohydrates, amino acids and the derivatives of carnitine (Supplementary Table S1). In Ch *B.dorsalis* flies, the metabolites at relatively higher levels mainly included free fatty acids like phytanic acid, linoleic acid and docosanoic acid. A total of 14 phospholipid compounds and 11 fatty acids compounds were identified among the differentially-accumulated metabolites, suggesting that lipids may play an important role in the blockage of RNAi in *B. dorsalis*.

### An increased ratio of LA to AA in the Ch flies down-regulates the extent of the endocytosis of dsRNA

**Figure 2:**
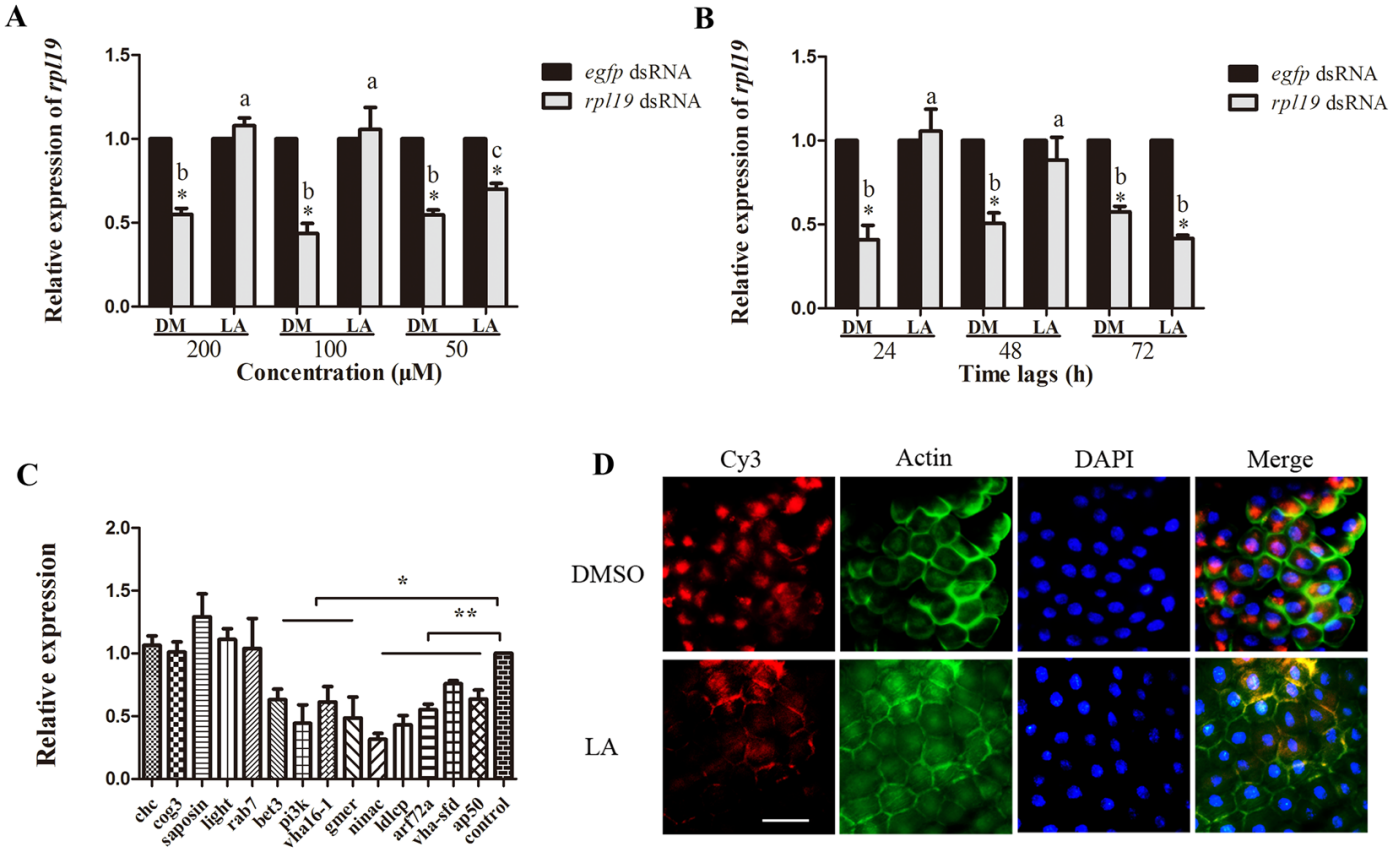
The blockage of RNAi caused by injecting linoleic acid. **(A)** mRNA accumulation of the target gene after feeding ds*-rpl19* followed by injection of LA at different concentrations. **(B)** Feeding with dsRNA at 24 h, 48 h and 72 h after injecting LA, the mRNA accumulation of the *rpl19*. **(C)** mRNA accumulation levels of genes known to be required for the endocytic entry of dsRNAs after injecting LA. **(D)** Subcellular localization of Cy3-labelled ds-*rpl19* in midgut tissue after the injection of LA into *B. dorsalis*. The scale bar represents 25 μm. Normalized target gene mRNA accumulation is reported relative to mRNA accumulation of the ds-*egfp* control, which was set to 1. All error bars represent the S.E. of the mean of three independent biological replicates. * indicates a statistically significant difference in *rpl19* mRNA accumulation between the feeding ds-*rpl19* group and the control ds-*egfp* group (P < 0.05). ** indicates P < 0.01 (Student’s t-test). Different letters indicate a significant difference in *rpl19* mRNA accumulation among the feeding ds-*rpl19* treatments (P < 0.05, Duncan’s test).

In our initial experiments for this part of the study, each of the up-regulated metabolites of the Ch group was injected into untreated flies to test its influence on RNAi. We found that only the injection of linoleic acid could induce a similar blockage of RNAi with that seen for the injection of the hemolymph from the Ch group (Fig. 2A,B, Supplementary Figure S3A). Injecting either 100 μM or 200 μM LA could completely block RNAi as induced by feeding. However, the mRNA accumulation of *rpl19* decreased only 30% after feeding ds*-rpl19* following injection of 50 μM LA while there was a decrease of 50% in control flies injected with the vehicle, 0.1% DMSO (DM) (Fig. 2A). Furthermore, after injecting 200 μM LA, the RNAi blocking effect lasted for 2 days (Fig. 2B). These results indicate that the RNAi blocking effect induced by LA is related to both concentration and time. We next tested the mRNA accumulation of genes that have been reported to be responsible for the cellular entry of dsRNA^9^. These genes are involved in several crucial steps of endocytosis, including vesicle formation and transport, intracellular transport, and lipid metabolism. qPCR analysis indicated that the mRNA accumulation of most of these genes was reduced, relative to controls, in flies injected with 200 μM LA and sampled 24 h after exposure to dsRNA (Fig. 2C). For example, the mRNA accumulation of *nina c*, which plays an important role in actin polymerization and cytoskeletal organization, was reduced by 65% in flies injected with 200 μM LA, as compared to controls. A similar magnitude of reduction was observed for *ldlCp* and *bet 3*, which are members of the *Golgi complex* (COG) family^9^. Genes that function in proton transport, including *vha16-1* and *vha-sfd*, also showed significant down-regulation in the flies injected with 200 μM LA. It was also the case that the mRNA accumulation of *arf72a* and *ap50* (involved in vesicle-mediated transport) and the lipid metabolism genes *pi3k* and *gmer* was decreased. Cy3-labelled dsRNA molecules were used to track the presence of dsRNA in midgut cells of insects injected with either LA or vehicle (Fig. 2D). Fluorescence microscopy revealed that dsRNA accumulated in a location near the nucleus in flies injected with the vehicle, while no dsRNAs were detected in the midgut cells of insects injected with LA, indicating an a lack of dsRNA entry into these cells. These results demonstrate that the endocytosis of dsRNA is impaired in LA-injected *B. dorsalis* individuals.

**Figure 3:**
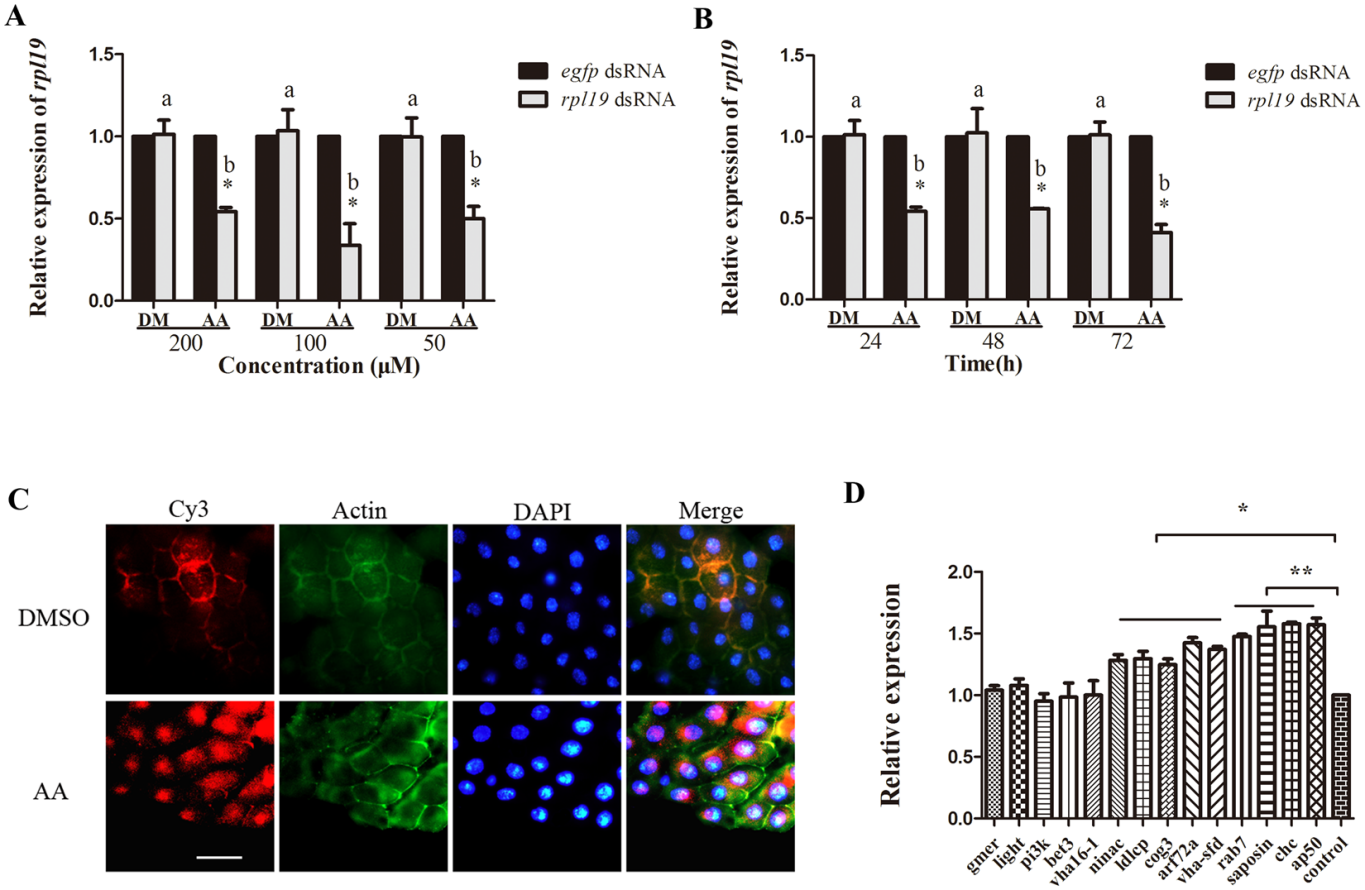
Rescue the RNAi effect in Ch flies by injectiing arachidonic acid. **(A)** mRNA accumulation of the target gene after feeding ds-*rpl19* in flies from the Ch group followed by injection of AA at different concentrations. **(B)** mRNA accumulation of the target gene in flies from the Ch group after feeding ds-*rpl19* for different time durations followed by injection of AA. **(C)** Subcellular localization of Cy3-labelled ds-*rpl19* in midgut tissue of *B. dorsalis* after injecting AA into the Ch flies. The scale bar represents 25 μm. **(D)** Expression levels of genes required for the endocytic entry of dsRNAs after injecting AA. Normalized target gene mRNA accumulation is reported relative to the mRNA accumulation in the ds-*egfp* control, which was set to 1. All error bars represent the S.E. of the mean of three independent biological replicates. * indicates a statistically significant difference in *rpl19* mRNA accumulation between the feeding ds-*rpl19* group and the control ds-*egfp* group (P < 0.05). ** indicates P < 0.01 (Student’s t-test). Different letters indicate a significant difference in *rpl19* mRNA accumulation among the feeding ds-*rpl19* treatments (P < 0.05, Duncan’s test).

We found that the LA:AA ratio in the hemolymph of the Ch group was 11.5:1, which was significantly higher than the 9.8:1 ratio observed for the Nv group (Supplementary Figure S3B). This result indicates that the relative content of AA to LA was decreased in the hemolymph of the Ch group. To test the role of AA in the blockage of RNAi in *B.dorsalis*, we injected AA into Ch group flies prior to a secondary exposure to ds-*rpl19*, to determine if the presence of AA could eliminate or otherwise alter the blockage of RNAi in the Ch group. Injection of AA could rescue the RNAi effect at all three concentrations tested (Fig. 3A). After injecting 200 μM AA, the recovery of the RNAi effect lasted for at least 3 days (Fig. 3B). Fluorescence microscopy analysis indicated that Cy3-labelled dsRNA could enter the midgut cells of flies in the Ch group following the injection of AA (Fig. 3C). The mRNA accumulation of genes involved in the cellular entry of dsRNA was also monitored; and most were found to be increased in AA-injecting flies as compared to DM-injecting flies up-regulated. For example, the *clathrin heavy chain* gene (*chc*), which is required for clathrin-mediated endocytosis, was up-regulated by 50%. A similar level of up-regulation was observed for the *saposin* and *ap50* genes. Other genes, including *ldlCp* and *cog3* (COG family), as well as *nina c*, *arf 72a*, and *rab7*, were also up-regulated (Fig. 3D).

### The fatty acid biosynthesis and metabolism pathways play important roles in the endocytosis of dsRNA in insects

Transcriptomic and proteomic analysis using RNA-seq and isobaric tags for relative and absolute quantitation (iTRAQ) on mixed samples from 8, 12, and 24 h (early samples) post primary exposure to dsRNA and samples from 5 days (late samples) post primary exposure to dsRNA were employed to identify differentially-expressed genes. Part of the transcriptome data of early samples has already been publised in our previous research and at that time we focused on the genes whose expression varied more than 2.5 times between Nv and Ch flies^10^. Here, we reanalyzed the transcriptome data of early samples with taking all the differentially expressed genes into consideration. In the transcriptome data of early samples, a total of 6,512,317 and 5,973,846 raw paired-end reads were identified for the Nv and Ch flies, respectively. In the late samples, 6,935,363 and 5,706,874 raw paired-end reads were identified for the Nv and Ch flies, respectively. After omitting the low-scoring sequenced reads, the average length of the clean reads was 100 bp. The total numbers of clean-reads in the early Nv and Ch libraries were, respectively, 6,363,092 and 5,793,444. In the late samples, 6,738,861 and 5,613,056 clean reads were obtained in the Nv and Ch libraries, respectively (Supplementary Table S2). We identified 2547 differentially expressed genes (DEGs) between Nv and Ch early samples and 1963 differentially expressed in late samples. There were 1204 up-regulated DEGs and 1343 down-regulated DEGs in the Ch flies as compared with the Nv flies in the early samples results (Supplementary Table S3). In the late samples, there were 844 up-regulated DEGs and 1119 down-regulated DEGs in the Ch flies as compared with the Nv flies (Supplementary Table S4). The RNA-seq data from this article are available as raw short read data in the National Center for Biotechnology Information’s Sequence Read Archive under accession number SRP075856. For the proteome results, the total number of peptide spectral features detected in the *B. dorsalis* samples was 323,953. After data filtering to exclude low-scoring spectra, 45,745 unique spectra that matched to particular peptides were obtained, and a total of 5,358 proteins were identified among all of the samples (Supplementary Table S5). In the early samples, a total of 115 differently expressed proteins, including 72 up-regulated and 43 down-regulated proteins, were identified in Ch flies compared with Nv flies (Supplementary Table S6–S7). In the late sample, 68 proteins were up-regulated and 79 proteins were down-regulated in the Ch flies as compared to the Nv flies (Supplementary Table S8–S9). Mass spectrometry data were deposited in the ProteomeExchange database under accession number PXD004272.

**Figure 4:**
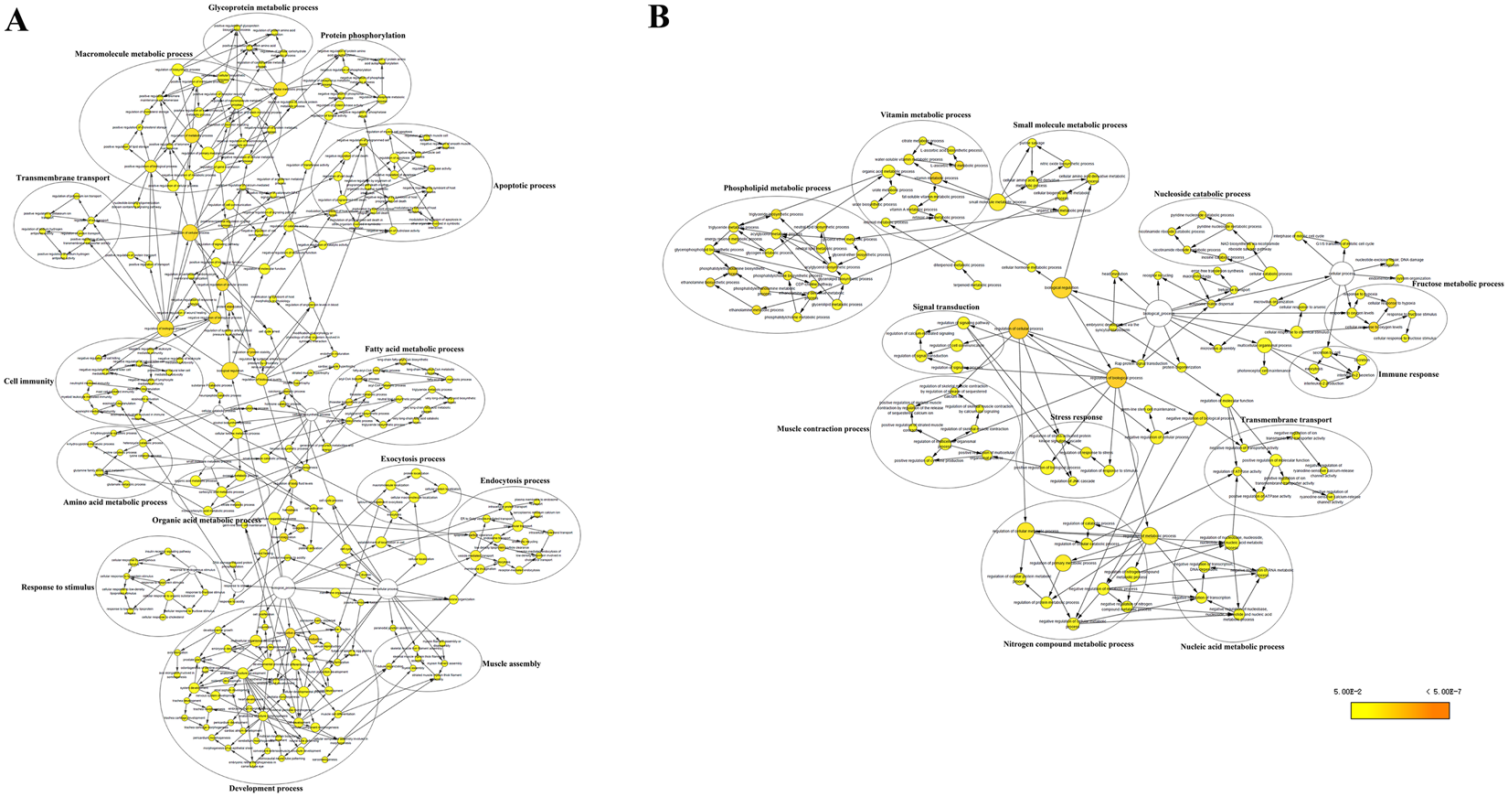
GO term overrepresentation analysis of biological processes. **(A)** Overrepresentation analysis of genes differentially expressed in the early samples. **(B)** Overrepresentation analysis of genes differentially expressed in the late samples. Nodes represent enriched GO terms. Edges connecting nodes indicate hierarchies and relationships between terms. Node size is proportional to the number of genes and proteins belonging to the functional category. Node color indicates the corrected P value for the enrichment of the term, according to the color legend. P < 0.05 was considered to be significant.

**Figure 5:**
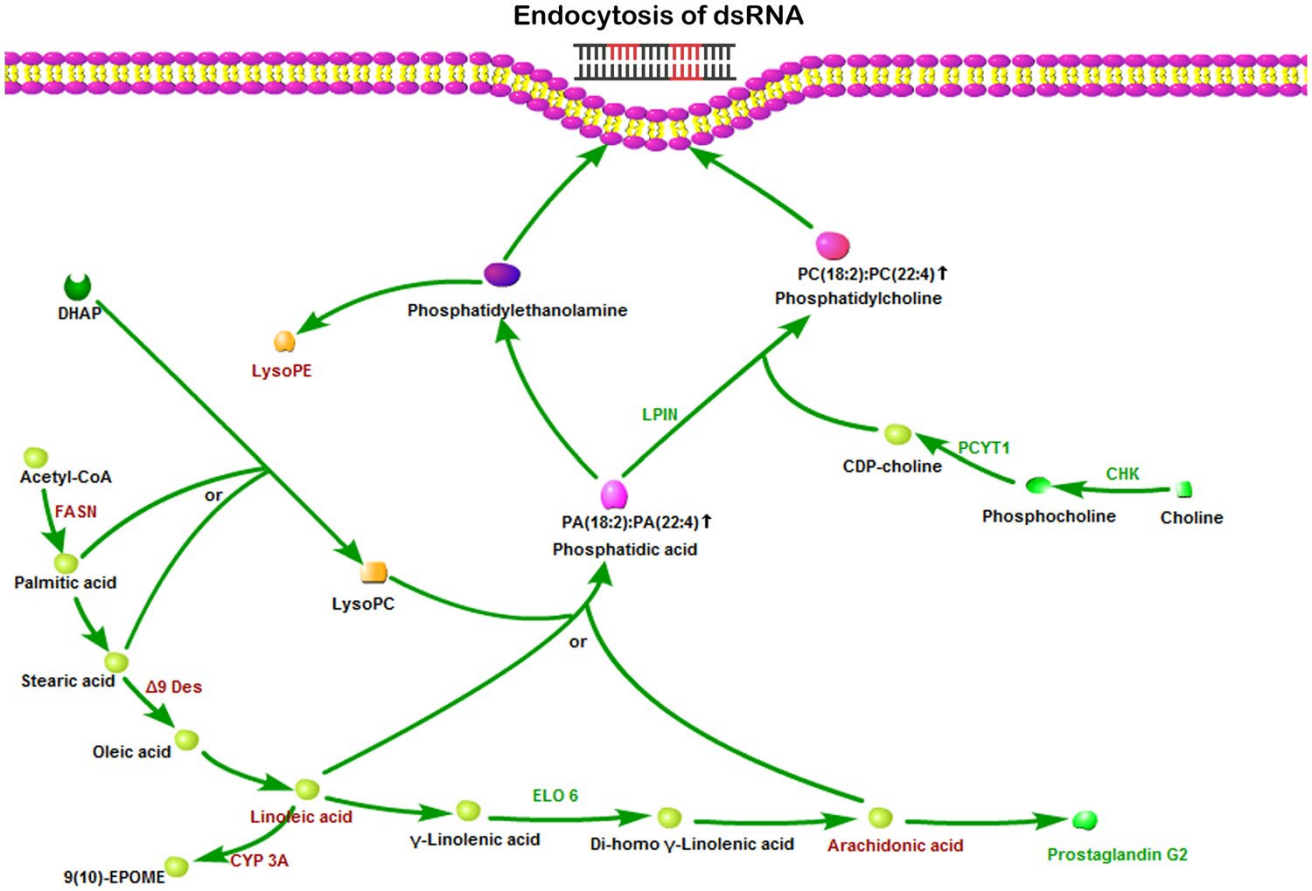
Schematic map of fatty acid biosynthesis and metabolism. Acetyl-CoA is the substrate for the the biosynthesis of plamitic acid, which is the precursor stearic acid and other fatty acids with longer carbon chains. Strearic acid can transform into oleic acid and LA after desaturation. LA is a precursor of AA. Both LA and AA can be esterified into phosphatidic acids which are the main substrates for the biosynthesis of the major cell membrane components, phosphatidylcholine and phosphatidylethanolamine. Notes in red and green colors denote up- or down-regulated genes, proteins and metabolites in the Ch group vs the Nv group, respectively.

GO enrichment analysis was used to examine the functional distribution of these genes and proteins. The combined transcriptomic and proteomic analyses results had 265 GO terms that were overrepresented in the early set samples and 132 GO terms were overrepresented in the late samples in the biological processes category (P < 0.05) (Fig. 4). GO terms overrepresented in the early set were mainly classified into 14 clusters; the overrepresented GO terms were mainly classified into12 clusters in the late samples. The ‘fatty acid metabolic process’ term was one of the major clusters overrepresented in the early set samples (Fig. 4A). The ‘phospholipid metabolic process’ term was the largest cluster in late samples (Fig. 4B). Based on previous research^18, 19^, our results for the metabolomics, transcriptomics, and proteomics analyses, we hypothesize (see Fig. 5) that the fatty acid biosynthetic and metabolic pathways may play an important role in the endocytosis of dsRNA into *B. dorsalis* midgut cells.

Brifely, fatty acid synthase (FASN) catalyzes the biosynthesis of palmitate by using acetyl-coenzyme A (acetyl-CoA) as substrate in a reductive reaction. Plamitic acid is the precursor stearic acid and other fatty acids with longer carbon chains. After desaturation, strearic transform into oleic acid and linoleic acid. Under the catalyzing of different fatty acid desaturases and elongases, LA can produce AA, which is the precursor of eicosanoids like prostaglandins. LA and AA can be esterified into phosphatidic acid separately. Phosphatidic acids are the main substrates for the biosynthesis of phosphatidylcholine and phosphatidylethanolamine, both of which are the major components of cell membranes. In this way, *B.dorsalis* could change the lipid compositions of cell membrane through the fatty acid biosynthetic and metabolic pathways to influence the endocytic ability to dsRNA in the midgut cells (Fig. 5).

### Silencing *fasn* restores the RNAi effect in the Ch *B.dorsalis*

**Figure 6:**
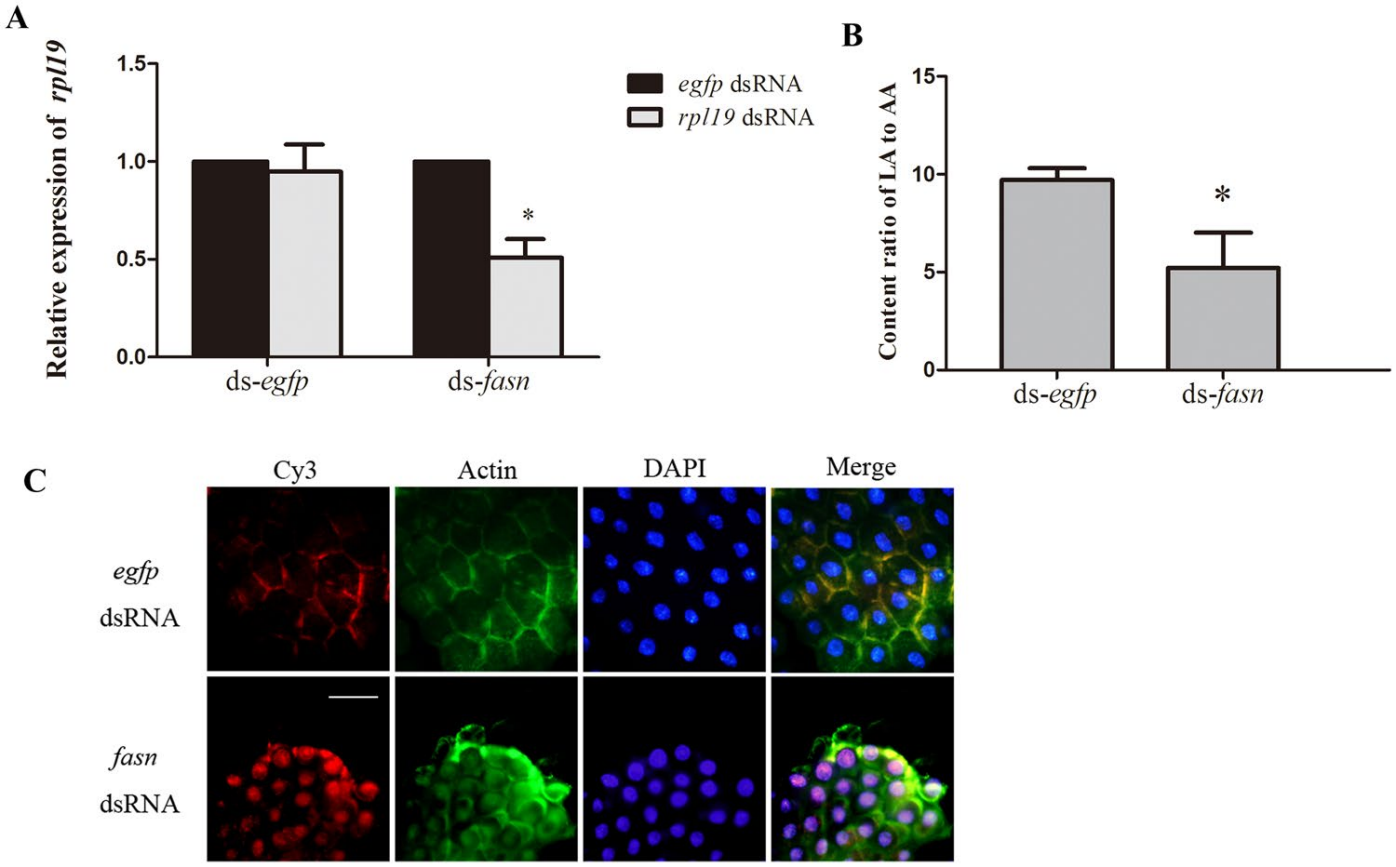
Effect of silencing *fasn* on the endocytosis of dsRNA. **(A)** mRNA accumulation of the target gene after secondary exposure in the *fasn*-silenced Ch flies. **(B)** The relative content ratio of LA:AA after the initial exposure to ds-*rpl19* followed by ds-*fasn* injection. **(C)** Subcellular localization of Cy3-labelled ds-*rpl19* in midgut tissue of *fasn*-silenced Ch *B. dorsalis* flies. The scale bar represents 25 μm. Normalized target mRNA accumulation is reported relative to mRNA accumulation in the ds-*egfp* control, which was set to 1. All error bars represent the S.E. of the mean of three independent biological replicates. * indicates a statistically significant difference in *rpl19* mRNA accumulation between the feeding ds-*rpl19* group and the control ds-*egfp* group (P < 0.05, Student’s t-test).

According to our hypothesis (Fig. 5), FASN is a key enzyme that mediates the endocytosis of dsRNA; it catalyses the reductive synthesis of long-chain fatty acids from acetyl-CoA and malonyl-CoA^20^. To test our hypothesis, we tested the function of *fasn* in blocking RNAi in *B. dorsalis* by using an ‘RNAi of RNAi’^21, 22^ approach. First, we confirmed that the expression of *fasn* was up-regulated within 5 days after the initial exposure, which provided the first evidence that *fasn* is involved in the endocytosis to dsRNA (Supplementary Figure S4A). Next, we tested the influence of the silencing of *fasn* on the blocking of RNAi in the Ch group samples. Injecting ds-*fasn* could induce down-regulation of target gene at least for 5 days (Supplementary Figure S4B). Based on this result, we performed the experiment that injecting ds-*fasn* before first exposure to ds-*rpl19* (Supplementary Figure S4C). The result indicated that the mRNA level of *fasn* was significantly decreased 5 days after first exposure to ds-*rpl19* in the ds-*fasn* injected flies (Supplementary Figure S4D). The mRNA accumulation of *rpl19* was down-regulated by 50% in the ds-*fasn* injected flies, while there was no difference in the mRNA accumulation of *rpl1*9 in the ds-*egfp* injected flies (control) following the secondary exposure, suggesting that silencing of the *fasn* gene can restore the RNAi effect in Ch *B. dorsalis* (Fig. 6A). Further testing showed that the ratio of LA:AA decreased to 4.88:1 in the ds-*fasn* injected flies but the number was 9.71:1 in the ds-*egfp* injected flies 5 days post primary exposure (Fig. 6B). Fluorescence microscopy analysis also indicated that dsRNA entered the midgut cells in *fasn*-silencing flies by endocytosis (Fig. 6C). These results confirm the supposition that *fasn* plays an important role in the loss of endocytic ability for dsRNA in *B. dorsalis* by altering the biosynthesis and metabolism of fatty acids.

### Injection of AA into *D. melanogaster* facilitate the uptake of ingested dsRNA

**Figure 7:**
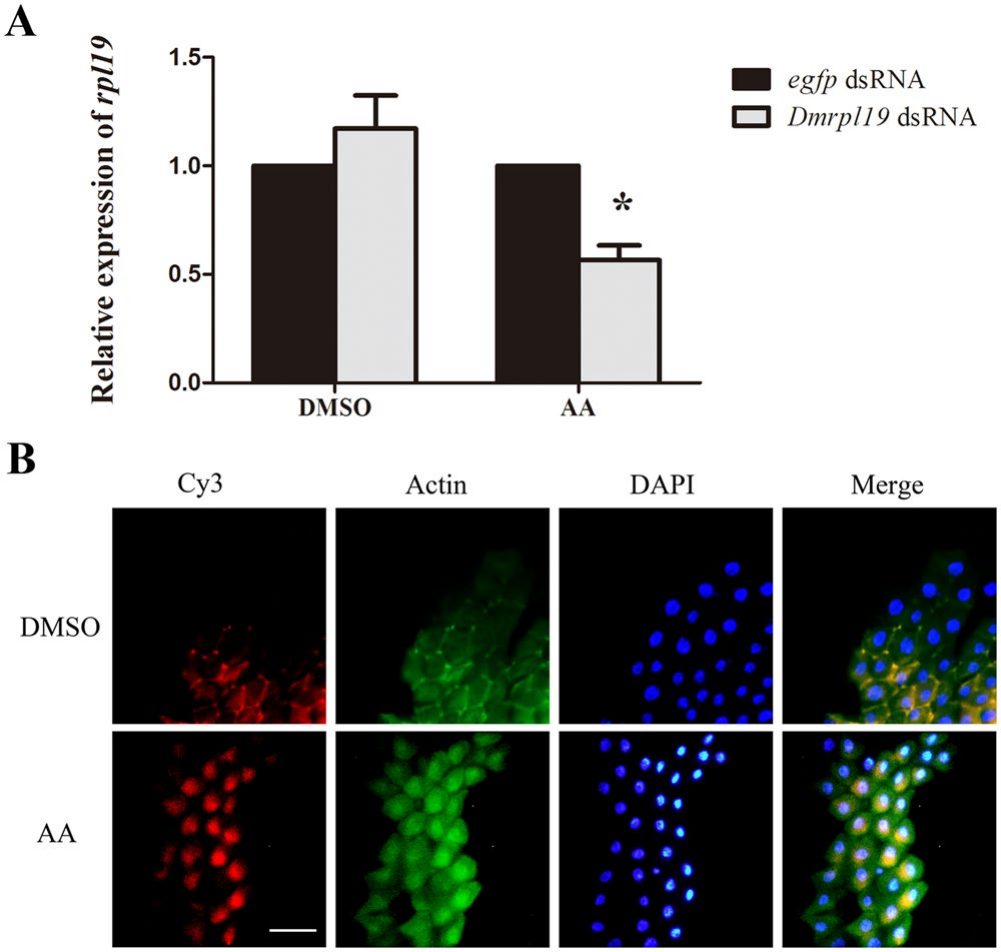
The influence of AA injection on the RNAi effect induced by the feeding of dsRNA in *D. melanogaster*. **(A)** mRNA accumulation of the target gene after feeding ds-*Dmrpl19* followed by injecting AA. (**B**) Subcellular localization of Cy3-labelled ds-*Dmrpl19* in midgut tissue of *D*. *melanogaster* after the injection of AA. The scale bar represents 25 μm. Normalized target gene mRNA accumulation is reported relative to mRNA accumulation in the ds-*egfp* control, which was set to 1. All error bars represent the S.E. of the mean of three independent biological replicates. * indicates a statistically significant difference in *rpl19* mRNA accumulation between the feeding ds-*rpl19* group and the control ds-*egfp* group (P < 0.05, Student’s t-test).

Previous research has demonstrated that feeding dsRNA to Drosophila does not induce an RNAi effect^12^. To test whether polyunsaturated fatty acids can also influence RNAi in Drosophila, we determined whether feeding dsRNA could produce RNAi effects in Drosophila after altering the LA:AA ratio via the injection of AA. Guided by our experience with *B. dorsalis*, AA was injected into Drosophila flies before letting the Drosophila flies feed on ds-*rpl19* from *D. melanogaster* (ds-*Dmrpl19*). qPCR analysis showed that feeding ds-*Dmrpl19* could induce a decrease of 44% to the mRNA accumulation of the *Dmrpl19* in AA-injected fruit flies (Fig. 7A). Cy3-labelled dsRNA was accumulated in a spot near the nucleus, indicating that AA injection facilitated dsRNA entry into midgut cells to produce RNAi effects in *D. melanogaster* (Fig. 7B).

## Discussion

The hemolymph injecting experiments support our hypothesis that there may exist an “RNAi blocking factor” in the hemolymph from Ch flies. In insects, the hemolymph acts as a blood-like carrier system delivering oxygen, carbohydrates, hormones, and proteins to the organs in the whole body^23^. Transfering hemolymph may cause physiological effect in recipient insects, for example, transfusing the hemolymph of post-critical weight donor can redce the development time of pre-critical weight larvae of *Manduca sexta* ^24^.

Fatty acids, especially PUFAs, are known to affect many cellular and physiological processes in both plants and animals, including endocytosis^18^. According to our data, in the Ch flies, dsRNA priming resulted in increases to the length and degree of unsaturation of the fatty acyl chains. Increasing the LA to AA ration resulted in the down-regulation of the endocytosis of dsRNA in midgut cells of Ch flies, and led to the blockage of RNAi in the secondary exposure to dsRNA. This result is consistent with previous findings. For example, in eukaryotes, the fluidity, flexibility, and selective permeability of membrane bilayers are known to be modulated by PUFAs^18^. Membrane lipids are key determinants of membrane curvature^25^. The longer and more unsaturated the acyl chains are in the phospholipids, the better can they facilitate endocytosis; polyunsaturated PLs have an effect on the activity of the GTPase dynamin and on the banana-shaped protein endophilin, which both play important roles in membrane fission^26^. Furthermore, changing the fatty acid composition of phospholipids can influence a number of cell signaling molecules^27^. So, there exists the possibility that altering the LA:AA ratio may affect some signal transduction pathways that mediate endocytosis of dsRNA in the Ch flies. In the present research, we changed the LA:AA ratio by injection. However, the result of UPLC/MS in our research was the relative content of each metabolites. In this way we just injecting a high concentrations of LA and AA to check the influence on RNAi blockage. In the subsequent research, we will explore the physiological concentration of each fatty acid since it will be helpful to further illuminate the mechanism mediating the RNAi blockage in *B.dorsalis*.

dsRNA can act as a trigger to silence endogenous gene expression. Similarly, in mammalian cells, some RNA viruses, like the vesicular stomatitis virus (VSV), can inhibit host gene expression. VSV produces matrix proteins that are located in both the cytoplasm and nucleus of infected cells; this matrix protein functions in the inhibition of host gene expression^28^. To defend against VSV infection, membrane-modifying oxysterols induced by interferons (IFN) in mammalian cells inhibit viral entry^29^. It’s widely accepted that invertebrates lack the IFN responses^30^. Considered in the context of previous studies our results suggest that another immune mechanism may exist in invertebrates to inhibit the entry of exogenous nucleic acids that may influence gene expression in a host.

Previous reports have demonstrated that dsRNA can enhance the antiviral ability of invertebrates. Injection of dsRNAs derived from vertebrate immunoglobulin genes, fish non-coding genomic DNA, bacterial vector sequences, and the Taura syndrome virus into marine shrimp evoked protection against infection with the white spot syndrome virus (WSSV) by activating some novel molecular mechanisms of innate immunity^30^. In Drosophila, host defenses against virus infection occur via the cleavage of viral dsRNA by Dicer-2^31^. Combining the results of these studies with our results, it is reasonable to hypothesize that the loss of endocytic ability induced by dsRNA in *B.dorsalis* may be an immune-like response that may be mediated by PUFAs. This response may defend against the threat caused by exogenous nucleic acids, such as viruses, that can cause abnormal gene expression in host cells. However, clearly the antiviral responses induced by dsRNA in insects need to be explored further and characterized in greater depth.

## Methods

### Insects

*B. dorsalis* was reared as described by Li^32^. Adult flies were maintained at 28 °C, with a 12 h light:12 h dark photoperiod, and were given an artificial diet consisting of 2.5% yeast extract, 7.5% sugar, 2.5% honey, 0.5% agar, and 87% H_2_O. Eggs and larvae were cultured using bananas.

### dsRNA preparation

The l4440 plasmid containing *egfp* and the target gene fragments was transformed into *Escherichia coli* HT115 (DE3) competent cells. Modified plasmids were constructed as described by Li^32^. After transformation, single colonies of HT115 (DE3) were cultured overnight in LB at 37 °C with shaking at 220 rpm. The culture was diluted 100-fold in 800 ml 2 × YT supplemented with 75 mg/ml ampicillin plus 12.5 mg/ml tetracycline, and cultured at 37 °C until reaching an OD600 value of 0.5. dsRNA synthesis by T7 polymerase was induced by adding 0.4 mM IPTG, and the bacteria were incubated with shaking for an additional 4 h at 37 °C.

Total nucleic acids were extracted as described by Timmons^33^. Samples were treated with RQ1 RNase-free DNase (Promega, USA) and RNase A solution (Promega, USA) before measuring concentrations using a NanoDrop 1000 (Thermo, USA). dsRNA solutions were also loaded onto a 2% agarose gel, stained with ethidium bromide, and photographed.

### Microinjection

Linoleic acid and arachidonic acid (Sigma-Aldrich, USA) were dissolved in 0.1% DMSO. Microinjection was performed using an InjectMan NI2 instrument (Eppendorf, Germany) equipped with a FemtoJet microinjection system. The glass capillaries used for microinjection were made from 50 µl glass micropipettes using a Puller at heater level 60.4 (PC-10, Narishige, Japan). The injection conditions were set to a Pi of 570 hpa and a Ti of 0.2 s. A total of 200 nl of solution was injected into each *B. dorsalis* fly^34^.

### Feeding bioassay

Flies emerged within 5 days and were collected and moved into a 17 cm × 8 cm × 7 cm box. Each treatment contained 50 flies (sex ratio 1:1) that were dehydrated and starved for 24 h. The artificial diet material was cut into circular pieces 3.2 cm in diameter and submerged in 800 μl of a dsRNA solution. The flies were fed the artificial diet supplemented with dsRNA starting at 8:00 am and were returned to a normal artificial diet at 14:00 pm the same day. The concentration of the dsRNA solution for the primary exposure was 10 ng/μl, and the concentration for the second exposure was 300 ng/μl^10^.

### Hemolymph preparation

Two different size Eppendorf centrifuge tubes were used to collect hemolymph at 5 days after primary exposure to dsRNA in a process using centrifugation at 2000 rpm/min^35^. Before injection, hemolymph samples were treated in two different ways. In one treatment, the hemolymph samples were incubated at 25 °C for 24 h, 48 h, and 72 h. In another treatment, the samples were incubated at −20 °C for 24 h, 25 °C for 24 h, and 100 °C for 10 min, and the hemolymph samples were then injected into the body cavities of untreated flies separately.

### Real-Time PCR

For each treatment, 10 flies (sex ratio 1:1) were collected for RNA extraction. RNA was extracted using RNAiso Plus reagent (Takara, Japan). cDNA was synthesized from 500 ng total RNA using Transcript RT Master Mix (Takara, Japan) following the manufacturer’s instructions. Real-time RT-PCR was performed using BioRad SYBR Green qPCR mix (BioRad, USA) on a BioRad MyIQ2 instrument. All RNA samples were analyzed in triplicate (tech reps). The reactions included 2 µl cDNA, 10 µl SYBR Green mix, 0.8 µl each of forward and reverse primers and 6.4 µl ddH2O. The thermocycler conditions were 95 °C for 30 s, followed by 40 cycles at 95 °C for 15 s and 60 °C for 30 s. Melting curve analysis was performed at the end of each expression analysis, using the following conditions: 55 °C for 60 s, followed by 81 cycles starting at 55 °C for 10 s with a 0.5 °C increase with each cycle^10^. The sequences of the qPCR primers used for the reference gene and the target genes were those described by Li^32^. The qPCR data were analyzed using the 2^−ΔΔCT^ method. The expression of *rpl19* and *fasn* was quantified relative to the levels of *rpl19* and *fasn* in the flies treated with *egfp* dsRNA. Triplicate biological experiments were performed independently. All results from experimental replicates were analysed using Student’s t-test or a one-way analysis of variance (ANOVA) and a Duncan’s test using SPSS 20 (IBM Corporation, USA).

### UPLC/MS analysis

Hemolymph samples were diluted with acetonitrile (v/v, 50 μL: 200 μL) for protein removal^36^. Ultra performance liquid chromatography (Waters Acquity BEH C18 column) coupled to a quadruple-time of flight mass spectrometer (Waters SYNAPT Q-TOF HDMS) was used for non-targeted metabolic profiling. The ion source was operated in both positive (ESI + ) and negative (ESI−) electrospray ionization modes (*i.e*., two separate analyses for each sample, in these separate modes). The P values was caculated by Student’s t-test.

### Immunofluorescence microscopy

A Silencer siRNA Labelling Kit with Cy3 (Ambion, USA) was used for fluorescent labelling of ds-*rpl19* following the manufacturer’s instructions. Midgut tissue was incubated with Cy3-labelled dsRNA for 1 h. Then, 4% formaldehyde was used to fix the tissue for 20 min. Actin was visualized with Acti-stainTM 488 fluorescent phalloidin (Cytoskeleton Inc., USA). Nuclei were counterstained with DAPI. Images were captured on an Olympus IX71 microscope (Olympus, Japan).

### RNAseq

Samples were collected 5 days after the first exposure. RNAseq was performed as described by Li^10^. After extraction, mRNA was purified using a Micropoly (A) Purist^TM^ mRNA purification kit (Ambion, USA) following the instruction manual. A SuperScript Double-stranded cDNA Synthesis Kit (Invitrogen, USA) was used for cDNA synthesis, and Ampure beads (Agencourt, USA) were used for purification. The purified cDNA was used to prepare a library using a TruSeq^TM^ DNA sample Prep Kit-Set A (Illumina, USA), and PCR amplification was performed using a TruSeq PE Cluster Kit (Illumina, USA). The products were sequenced on an Illumina HiSeqTM 2000 System (Illumina, USA), and clean reads were mapped to a *B. dorsalis* transcriptome dataset^37^. We then quantified transcript levels in reads per kilobase per million mapped reads (RPKM). The significance of differentially expressed genes was analysed using an MAplot-based method^38^.

### Isobaric Tag for Relative and Absolute Quantitation (iTRAQ)

Two sets of samples were collected. Mixed samples of whole flies were collected 8, 12, and 24 h after the primary exposure, and another samples was collected 5 days after the primary exposure. The procedures for the quantitative proteomics experiments that we performed were essentially those described by Wang^39^. Briefly, after a reductive alkylation reaction, proteins (100 μg) from each sample were digested with trypsin and labelled with 8-plex iTRAQ reagents (Applied Biosystems, CA). Labelled samples were pooled and resolved into 12 fractions using an Ultremex SCX column(Phenomenex, USA). The eluted fractions were then desalted. Analytical separation was performed using an LC-20AB liquid chromatograph (SHIMADZU, Japan) coupled with a Triple TOF 5600 MS instrument (AB SCIEX, USA) that was fitted with a Nanospray III source (AB SCIEX, USA). A pulled-quartz tip was used as the emitter (New Objectives, USA). Data were acquired using an ion spray voltage of 2.5 kV, curtain gas at 30 PSI, nebulizer gas at 15 PSI, and an interface heater temperature of 150 °C. Peptides were identified by searching against a previously-described database^37^ with an MS/MS data interpretation algorithm implemented in Mascot software (http://www.matrixscience.com). In this study, we used P < 0.05 and a fold change > 1.2-fold or <0.8-fold as the threshold to judge the significance level of differential protein expression.

### GO Term Overrepresentation Analysis of Biological Processes

Overrepresentation analysis of genes differentially expressed between the Nv and Ch groups, with respect to their GO terms describing biological processes, was analysed using the Cytoscape plug-in BiNGO 2.4^40^.

## Electronic supplementary material

Supplementary information

Dataset 1

Dataset 2

Dataset 3

Dataset 4

Dataset 5

Dataset 6
