# Supplementary material for: The inducible blockage of RNAi reveals a role for polyunsaturated fatty acids in the regulation of dsRNA-endocytic capacity in Bactrocera dorsalis
Source: Sci Rep. 2017 Jul 17;7:5584. doi: 10.1038/s41598-017-05971-0 (PMC5514080; doi:10.1038/s41598-017-05971-0)
Supplement: Supplementary file 1 — Supplementary information [file 41598_2017_5971_MOESM1_ESM.pdf]

Supplementary Information

The inducible blockage of RNAi reveals a role for polyunsaturated fatty acids in the regulation of dsRNA-endocytic capacity in *Bactrocera dorsalis*

Xiaolong Dong[1], Xiaoxue Li[1], Qiujia Li[1], Hongmei Jia[2] and Hongyu Zhang[1,*]

[1]State Key Laboratory of Agricultural Microbiology, Institute of Urban and Horticultural Entomology, College of Plant Science and Technology, Huazhong Agricultural University, Wuhan 430070, Hubei, People's Republic of China.

[2]Institute of Medicinal Plant Development, Chinese Academy of Medical Sciences and Peking Union Medical College, No. 151 Malianwa North Road, Haidian District, Beijing 100193, People's Republic of China.

[*] Correspondence : hongyu.zhang@mail.hzau.edu.cn

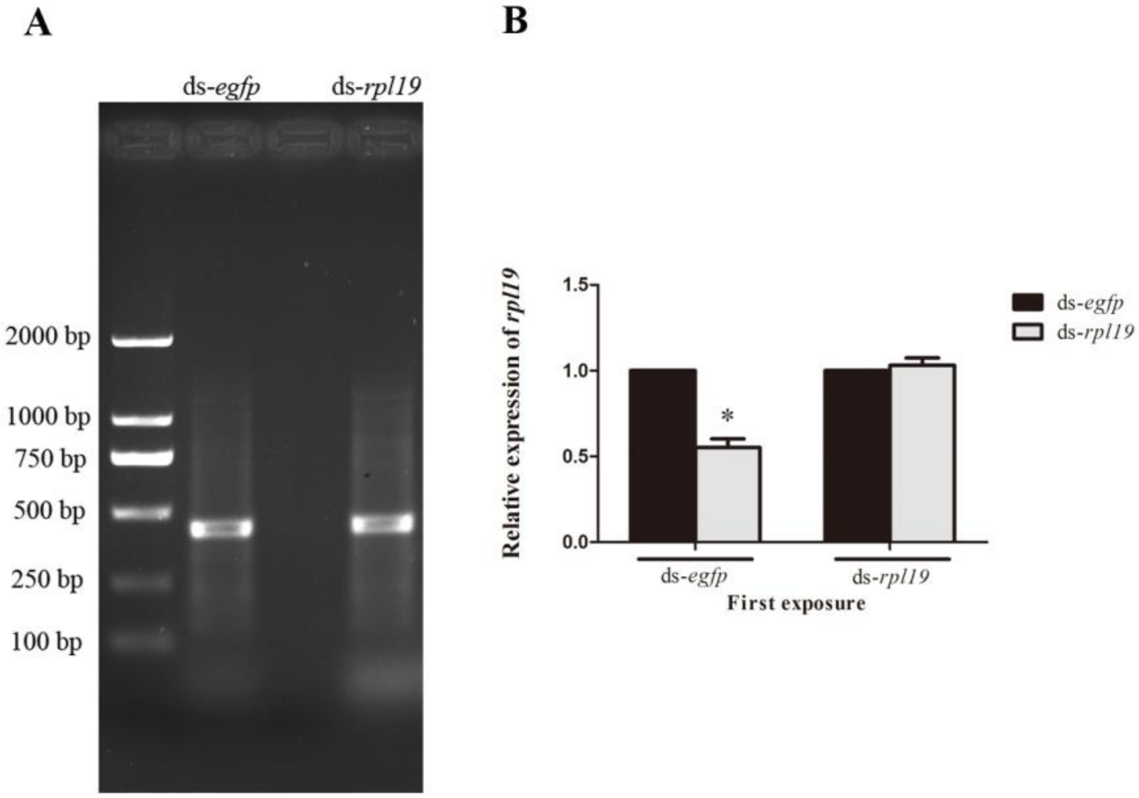

Figure S1 Inducing the blockage of RNAi in *B.dorsalis* by feeding with dsRNA. **(A)** The agarose gel electrophoresis of dsRNA applied in feeding experiments prepared by bacteria. **(B)** The blockage of RNAi in the secondary exposure to ds-*rpl19* induced by *in vitro* transcribed dsRNAs. Normalized target gene mRNA accumulation is reported relative to the mRNA accumulation in the ds-*egfp* control, which was set to 1. All error bars represent the S.E. of the mean of three independent biological replicates. * indicates a statistically significant difference in *rpl19* mRNA accumulation between the feeding ds-*rpl19* group and the control ds-*egfp* group ($P < 0.05$, Student's t-test).

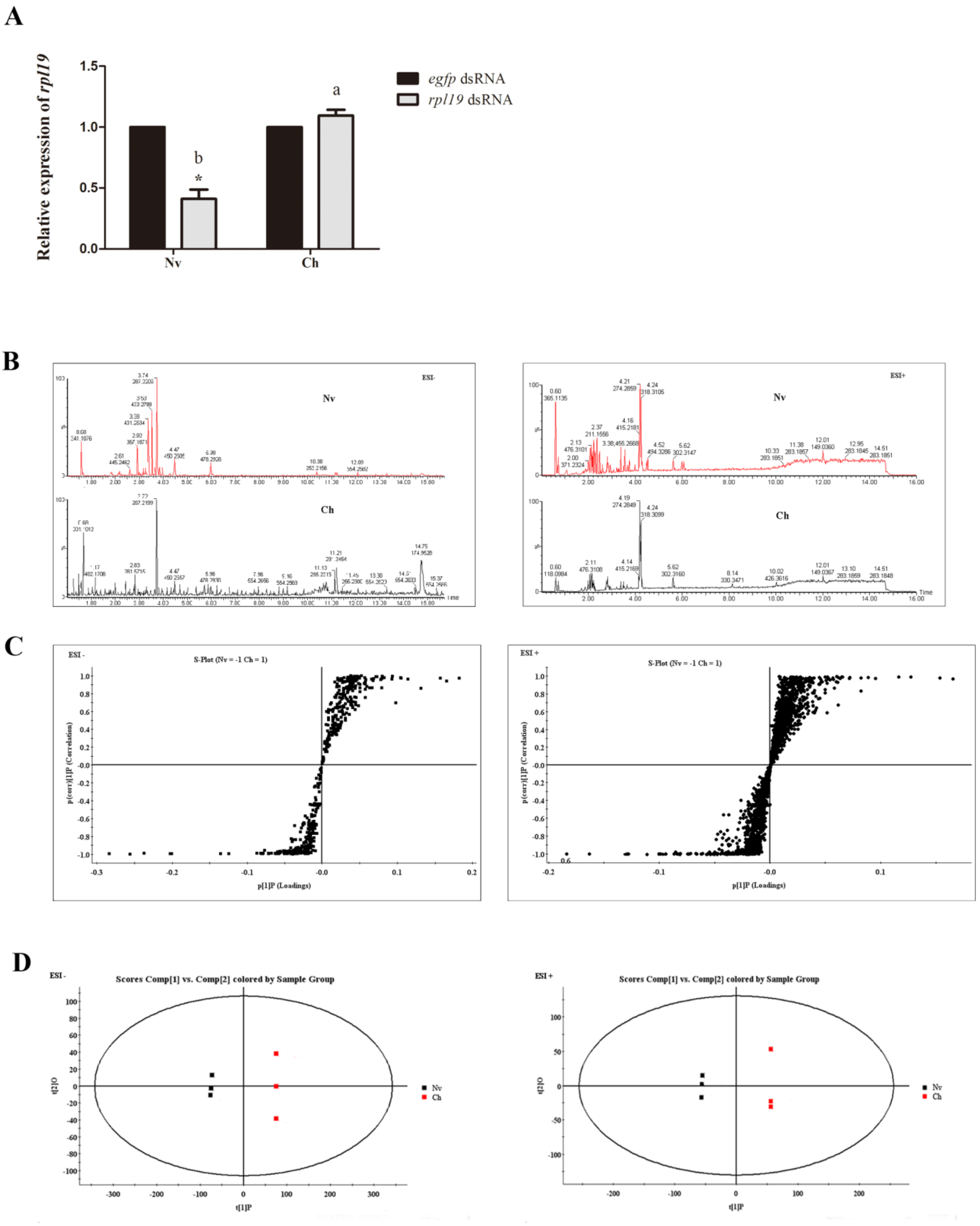

A
Relative expression of rpl19
1.5
1.0
0.5
0.0
Nv
Ch
b
*
a
egfp dsRNA
rpl19 dsRNA
B
ESI-
Nv
Ch
ESI+
Time
C
ESI -
S-Plot (Nv = -1 Ch = 1)
p(corr)[1]P (Correlation)
p[1]P (Loadings)
ESI +
S-Plot (Nv = -1 Ch = 1)
D
ESI -
Scores Comp[1] vs. Comp[2] colored by Sample Group
t[2]O
t[1]P
Nv
Ch
ESI +
Scores Comp[1] vs. Comp[2] colored by Sample Group

Figure S2 The total ion current (TIC) profiles, S-plot, and OPLS-DA scores plot of the Nv and Ch *B.dorsalis* hemolymph samples. **(A)** The blockage of RNAi in the secondary exposure after initial priming with ds-*rpl19*. **(B)** Total ion current (TIC) profiles of Hemolymph from Nv (upper) and Ch *B. dorsalis* (lower) samples (UPLC/MS, analyses in both ESI + and ESI – mode). **(C)** S-plots of the Ch and Nv hemolymph samples as analyzed in ESI + and ESI – mode. **(D)** OPLS-DA scores plot separating Ch (red squares) and Nv (black squares) samples. Normalized target mRNA levels are reported relative to level of the ds-*egfp* control, which was set to 1. All error bars represent the S.E. of the mean of three independent biological replicates. * indicates a statistically significant difference in *rpl19* expression between the feeding ds-*rpl19* group and the control ds-*egfp* group ($P < 0.05$, Student's t-test). Different letters indicate a significant difference in *rpl19* mRNA accumulation among the feeding ds-*rpl19* treatments ($P < 0.05$, Duncan's test).

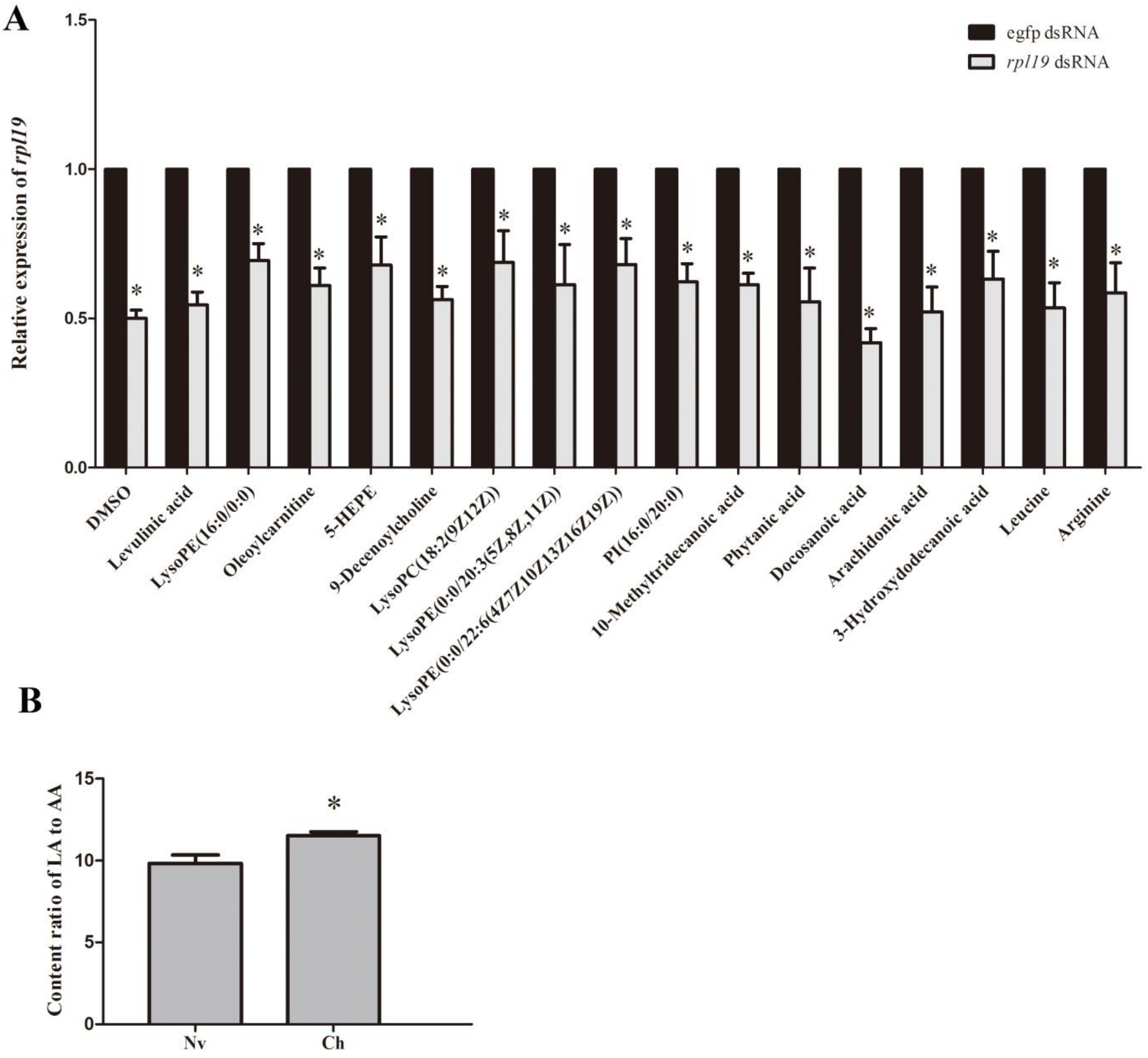

Figure S3 The influence of up-regulated metabolites in Ch group to RNAi effect of *B.dorsalis*. **(A)** Feeding with dsRNA after injecting the up-regulated metabolites identified in the Ch group, the mRNA accumulation of the *rpl19*. **(B)** The LA:AA ratio after initial priming with ds-*rpl19*. Normalised target gene accumulation is reported relative to accumulation of the mRNA of the ds-*egfp* control, which was set to 1. All error bars represent the S.E. of the mean of three independent biological replicates. * indicates a statistically significant difference in *fasn* mRNA accumulation between the feeding ds-*rpl19* group and the control ds-*egfp*, or between the injected ds-*fasn* group and the control ds-*egfp* ($P < 0.05$, Student's t-test).

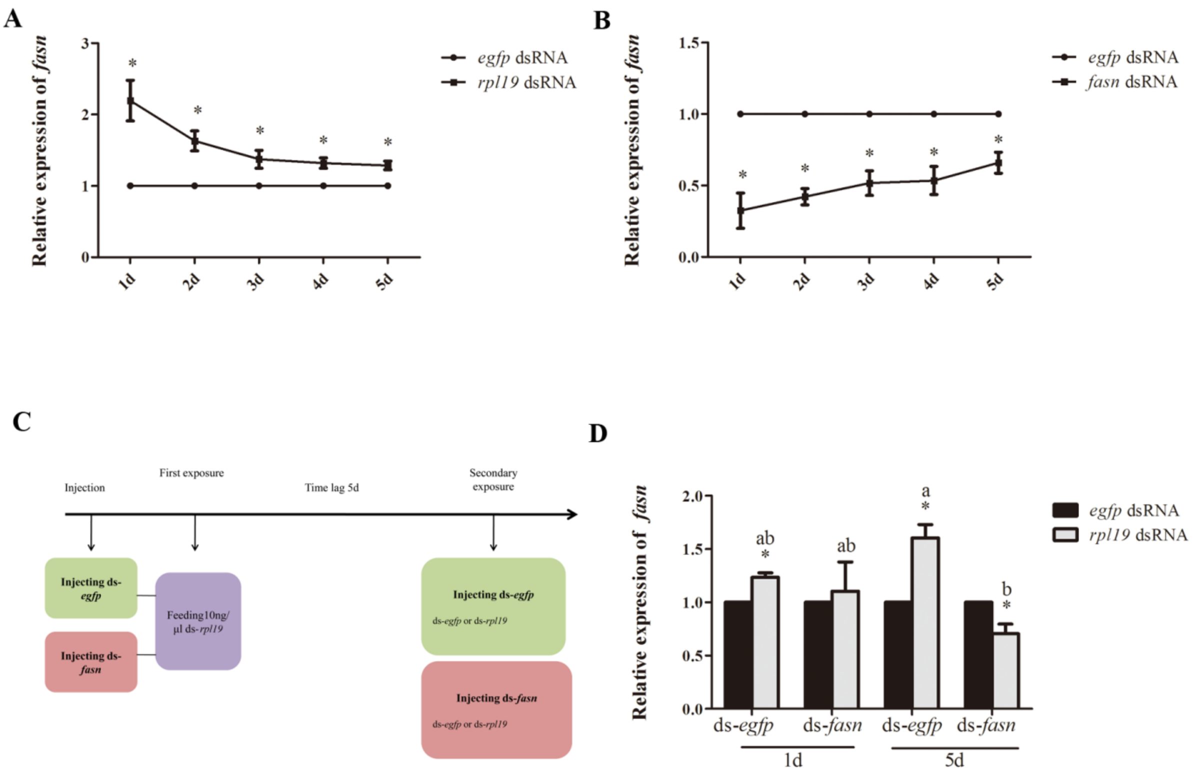

Figure S4 The influence of feeding dsRNA on the accumulation of mRNA of the *fasn* gene. **(A)** *fasn* mRNA accumulation after feeding on ds-*rpl19*. **(B)** Accumulation of mRNA of the *fasn* gene after injecting ds-*fasn*. **(C)** Schematic representation of the experimental design. The timeline depicts the experimental process, in days. After injecting dsRNA, the flies were fed with 10 ng/μl ds-*rpl19*. The ds-*egfp* injected group and the ds-*fasn* injected group were fed with ds-*egfp* or ds-*rpl19* during the secondary exposure period. **(D)** accumulation of mRNA of the expression of the *fasn* gene after feeding on ds-*rpl19*, followed by injection with ds-*fasn*. Normalised target gene accumulation is reported relative to accumulation of the mRNA of the ds-*egfp* control, which was set to 1. All error bars represent the S.E. of the mean of three independent biological replicates. * indicates a statistically significant difference in *fasn* mRNA accumulation between the feeding ds-*rpl19* group and the control ds-*egfp*, or between the injected ds-*fasn* group and the control ds-*egfp* ($P < 0.05$, Student's t-test). Different letters indicate a significant difference in *fasn* mRNA accumulation among the feeding ds-*rpl19* treatments ($P < 0.05$, Duncan's test).

Table S1

The list of 37 metabolic features that have different abundance between Nv and Ch haemolymph

	Metabolite	RT	M/Z	*P-value*	Content Ratio (Ch / Nv)
ESI-	Levulinic acid	2.02	116.0473	0.000	2.57 (up)
	Aspartylysine	2.14	261.275	0.000	0.17 (down)
	Prostaglandin G2	2.92	368.2198	0.000	0.04 (down)
	3-hydroxypentadecanoic acid	3.04	258.3969	0.002	0.12 (down)
	DG(18:2/22:6/0:0)	3.25	664.5066	0.001	0.51 (down)
	1-Palmitoylglycerophosphoinositol	3.65	572.2961	0.022	0.17 (down)
	Glucitol	4.24	318.3095	0.000	0.42 (down)
	LysoPE(16:0/0:0)	4.43	453.2855	0.001	1.45 (up)
	1-Oleoylglycerophosphoinositol	4.45	598.6604	0.000	0.02 (down)
	LysoPE(16:1)	4.46	451.5344	0.000	0.53 (down)
	LysoPC(16:1(9Z))	4.51	493.3168	0.000	0.03 (down)
	Lysopc(18:1)	6.07	522.3572	0.001	0.33 (down)
	Oleoylcarnitine	10.03	426.3628	0.000	1.24 (up)
	5-HEPE	10.41	318.2194	0.002	3.32 (up)
	9-Decenoylcholine	11.15	256.4042	0.000	1.75 (up)
	Stearic acid	0.6	341.1071	0.000	0.32 (down)
ESI+	Creatinine	1.07	155.0944	0.032	0.21 (down)
	PC(14:0/24:1(15Z))	2.15	280.2125	0.048	0.20 (down)
	Glutarylglycine	2.17	144.0933	0.000	0.09 (down)
	LysoPC(18:2(9Z,12Z))	2.17	520.3343	0.002	1.74 (up)
	PC(18:4(6Z,9Z,12Z,15Z)/24:1(15Z))	2.2	453.348	0.049	0.28 (down)
	LysoPE(0:0/20:3(5Z,8Z,11Z))	2.21	564.3581	0.02	1.39 (up)
	LysoPE(0:0/22:6(4Z,7Z,10Z,13Z,16Z,19Z))	2.25	608.3821	0.046	1.56 (up)
	PI(16:0/20:0)	2.76	456.2835	0.005	2.10 (up)
	Heptacosanoic acid	2.93	217.2069	0.006	0.36 (down)
	TG(14:0/18:4(6Z,9Z,12Z,15Z)/o-18:0)	3.26	279.2416	0.002	0.32 (down)
	10-Methyltridecanoic acid	3.5	246.2546	0.004	1.71 (up)
	Tricosanoic acid	3.54	219.2237	0.011	0.46 (down)
	Caprylic acid	3.74	311.2289	0.000	0.48 (down)
	LysoPE(16:1(9Z)/0:0)	4.48	452.2813	0.034	0.48 (down)
	Phytanic acid	8.14	330.3455	0.036	1.51 (up)
	Docosanoic acid	10.66	358.3748	0.001	1.72 (up)
	Linoleic acid	11.21	281.2459	0.000	1.56 (up)
	Arachidonic acid	11.46	327.2109	0.042	1.31 (up)
	3-Hydroxydodecanoic acid	12.39	239.1601	0.016	3.39 (up)
	Leucine	14.72	158.9761	0.000	1.35 (up)
	Arginine	14.74	174.9538	0.000	1.42 (up)

The single and double underlines represent compounds of fatty acids and phospholipids respectively.

Table S2

Summary of DGE metrics from *B. dorsalis* transcriptomes.

**Metrics**	**Number**			
	Early set		Late set	
	Nv	Ch	Nv	Ch
Total reads	6512317	5973846	6935363	5706874
Clean reads	6363092(97.7%)	5793444(96.9%)	6738861(97.1%)	5613056(98.3%)
Length	100	100	100	100

Table S5
Summary of iTRAQ metrics from *B. dorsalis* proteomes

**Metrics**	**Number**	
Total spectra	323953	
Unique spectra	45745	
Matched protein	5358	
Differentially expressed	Early set	Late set
protein	115	147
